# Public Health, Hygiene and Bacteriology

**Published:** 1895-10

**Authors:** Ernest Wende

**Affiliations:** Health Commissioner, Buffalo, N, Y.


### PUBLIC HEALTH, HYGIENE AND BACTERIOLOGY.

Conducted by ERNEST WENDE, M. D.,
Health Commissioner, Buffalo, N, Y.

### A Healthy Community.

By FRANKLIN C. GRAM, M. D., Registrar of Vital Statistics.

August, 1895, was the healthiest of any similar month in Buffalo of which we have any record. There were only 444 deaths, with a rate of 15.87, as against 550 for the same month in 1894, when, according to the city directory, there was a difference of 24,020 in the population. This is certainly a remarkable decrease, most of which occurred in children under one year of age, as shown by the following comparative table, the figures being for August, 1895, 1894, 1893 and 1892 respectively :

| | 1895. | 1894. | 1893. | 1892. |
|---|---|---|---|---|
| Under one year | 154 | 221 | 325 | 356 |
| One to two years | 49 | 61 | 91 | 68 |
| Two to three years | 16 | 12 | 28 | 21 |
| Three to four years | 10 | 9 | 6 | 12 |
| Four to five years | 4 | 7 | 12 | 5 |
| Totals under five years | 233 | 310 | 462 | 462 |

The deaths for August for the past five years have been as follows : 1891, deaths, 601, rate, 28.28 ; 1892, deaths, 712, rate, 29.97 ; 1893, deaths, 741, rate, 29.64 ; 1894, deaths, 550, rate, 20.95 ; 1895, deaths, 444, rate, 15.87.

For the adult ages, the difference is comparatively small. Of the total number, 251 were males, 193 females, 150 married and 294 single.

Cholera infantum is the disease which naturally heads the list at this period, having claimed 103 victims as against 126 for the same month last year. Eight persons died of diphtheria, two of them being adults of twenty and fifty years respectively. Two died of scarlet fever, one of measles, three of pertussis, nine of typhoid fever and thirty-eight of consumption. Of the remainder, twenty-one died of other communicable diseases; fifteen from senile debility; forty-two from perverted or deficient nutrition; fifty-two from diseases of the nervous system; of the circulatory system, twenty; respiratory system, twenty-seven; digestive system, sixty-seven; urinary system, nine; and violence, twenty-seven.

There were ninety-one cases of consumption reported, two of measles, fourteen of scarlet fever, forty-eight of diphtheria and sixty-five of typhoid.

According to the bulletin of the New York State Board of Health for July, which has just been issued, the mortality throughout the state was less than it has been for any July since 1891, the reported number of deaths for 1892, 1893 and 1894 having been respectively 13,555, 12,337 and 12,516, as against 11,681 this year. From diarrheal diseases there were 2,974 deaths, or more than one-fourth of the total deaths from all causes. In the six preceding months there were but 1,286 deaths from this cause, of which one-half occurred in June. One-half of the deaths from all causes occurred under the age of five years, which is below the average for July. Next comes consumption with 1,040 deaths.

The death-rate in some of the cities and towns of the state for July was: Buffalo, 19.08; Rochester, 19.60; Tonawanda, 20.00; North Tonawanda, 32.50; Lockport, 16.50; Niagara Falls, 16.50; Medina, 13.35; Albion, 18.20; Oswego, 10.00; Canandaigua, 21.00; Batavia, 19.92; Dansville, 19.15; Auburn, 15.50; Syracuse, 19.82; Dunkirk, 10.00; Jamestown, 11.00; Olean, 15.00; Hornellsville, 10.10; Elmira, 14.40; Binghamton, 14.25; Saratoga, 22.00; Waterford, 30.00; Rome, 12.00; Utica, 17.30; Schenectady, 18.90; Ogdensburg, 24.00; Watertown, 13.45; Troy, 23.07; Cohoes, 24.30; Albany, 20.25; Yonkers, 27.35; Long Island City, 30.75; Brooklyn, 26.85, and New York, 26.35.

Newton, a city with a population of 19,776, enjoys the distinction of having the highest death-rate of any part of the state—namely, 42.00; and Wappinger Falls, with 3,718 population, has the lowest of 8.00.

